# Mendelian randomization analysis reveals no causal relationship between thyroid function and sarcopenia-related traits

**DOI:** 10.3389/fendo.2024.1406165

**Published:** 2024-09-13

**Authors:** Rui Xu, Yan-Yan Li, Hong Xu

**Affiliations:** ^1^ Gerontology center, People’s Hospital of Xinjiang Uygur Autonomous Region, Urumqi, China; ^2^ Department of cardiac surgery, People’s Hospital of Xinjiang Uygur Autonomous Region, Urumqi, China

**Keywords:** Mendelian randomization, thyroid function, sarcopenia, hand-grip strength, appendicular lean mass, walking pace

## Abstract

**Background:**

Recent research has indicated a potential association between thyroid function and sarcopenia, but the specific mechanisms and a definitive causal relationship have yet to be established. Therefore, the objective of this study is to examine the potential causal connection between thyroid function and sarcopenia-related traits, including hand-grip strength, appendicular lean mass (ALM), and walking pace.

**Methods:**

The study used a bi-directional two-sample MR design, with thyroid function examined as the exposure and sarcopenia-related traits as the outcome in the first stage, and then reversed in the second stage. The genetic instruments for thyroid function were obtained from a comprehensive meta-analysis involving 271,040 participants. Data on sarcopenia-related traits based on GWASs were collected from the UK Biobank, which includes up to 461,026 European participants. The estimates for MR were calculated using the inverse-variance weighted (IVW) method, and several sensitivity analyses were performed.

**Results:**

After applying the Bonferroni correction for multiple testing, our MR analyses revealed no significant impact of thyroid function liability on sarcopenia-related traits. Similarly, our reverse MR analysis did not provide evidence supporting the influence of liability to sarcopenia-related traits on thyroid function. The results of the primary IVW MR analyses were largely in line with those obtained from our sensitivity MR analyses.

**Conclusion:**

Our research findings do not suggest a link between thyroid function and sarcopenia-related traits. The associations identified in epidemiological studies may be influenced, at least in part, by shared biological mechanisms or environmental confounders.

## Introduction

As the global population continues to age, the prevalence of sarcopenia is rising, leading to a decline in quality of life and increased mortality rates (1). According to the 2019 consensus update from the Asian Working Group for Sarcopenia (AWGS), the occurrence of sarcopenia in the elderly population is estimated to be between 6.8%-25.7% (2, 3). Sarcopenia is associated with an elevated risk of falls, recurrent falls, fractures, and mortality. Furthermore, it has been significantly linked to cardiometabolic disease and cognitive impairment (4, 5). Nevertheless, the precise etiology of sarcopenia remains incompletely elucidated and there are presently no efficacious pharmaceutical interventions for its management. A more comprehensive comprehension of the underlying mechanisms is imperative for advancing efforts in the prevention and treatment of this condition.

Previous studies have suggested that the pathogenesis of sarcopenia may involve various factors, including aging (6), neuromuscular dysfunction (7), mitochondrial dysfunction (8), proinflammatory cytokines (9), myocyte apoptosis (10), and genetic predisposition (11). Recent studies have indicated that thyroid disorders can result in a reduction of skeletal muscle mass and strength in mice, suggesting a correlation between both hypo- and hyperthyroidism with sarcopenia (12). In recent studies, associations have been identified between variations in normal-range thyroid function and the risk of sarcopenia in cohort studies (13). However, it remains uncertain whether these associations are causal, given that observational studies are susceptible to selection bias, residual confounding, and reverse causality. The regulation of intracellular T3 levels plays a critical role in the progression of myogenesis and contributes to the optimization of mitochondrial function in skeletal muscle (14). Several experimental studies have suggested that the supplementation of thyroid hormones in patients with hypothyroidism may potentially improve both muscle strength and cross-sectional area (15). However, the randomized controlled trials (RCTs) for these treatments have yielded conflicting results, and their limited sample sizes and/or brief follow-up durations preclude any definitive conclusions.

The studies mentioned collectively suggest a strong association between thyroid function and sarcopenia. However, it is crucial to ascertain the causality of the observed connections. MR is commonly employed to establish causality in scenarios where RCTs are not feasible or inaccessible (16). This approach leverages genetic variations as proxies to evaluate the direct influence of a factor (e.g. thyroid function) on the outcome of interest (e.g. sarcopenia) (17). The concept is grounded in the stochastic distribution of genetic variations from progenitors to descendants, akin to the randomization procedure employed in RCTs (18). Reversing the direction of causality, an observed association between the outcome under investigation and the genetically predicted exposure can provide support for a potential causal relationship, given that genetic variants have the potential to influence the outcome of interest.

During the course of this novel study, we conducted a two-sample MR to investigate the potential causal effects of variations in normal-range levels of free triiodothyronine (FT3), free thyroxine (FT4), total T3 (TT3), total T4 (TT4), thyrotropin (TSH), and FT3:FT4 ratio on sarcopenia-related traits. Additionally, reverse MR analyses were performed to assess the impact of sarcopenia-related traits on thyroid function.

## Materials and methods

### Two-sample Mendelian randomization

In this study, we employed a bi-directional MR study design and utilized two-Sample MR methodologies with various genome-wide association study (GWAS) summary level datasets to investigate the causal relationship and direction of causation between thyroid function (including FT3, FT4, TT3, TT4, TSH, and FT3:FT4 ratio) and sarcopenia-related traits (including hand-grip strength, ALM, and walking pace) in a European population. The study was conducted in two phases. Initially, an examination of the potential causal relationship between thyroid function and sarcopenia-related traits was undertaken. Subsequently, an assessment of the potential connection between genetic sarcopenia-related traits and thyroid function was carried out.

### Exposures (thyroid hormone levels) datasets

The exposures of interest were normal-range levels of FT3, FT4, TT3, TT4, TSH and FT3:FT4 ratio, which were from a recent GWAS on thyroid function in the ThyroidOmics Consortium (19). We identified single nucleotide polymorphisms (SNPs) that are independently associated with normal-range thyroid hormone levels at a genome-wide significant level (*p* < 5×10^-8^), with clumping *r* ^2^ = 0.001 and kb = 10,000, respectively (20). We employed the identified genetic variations as instruments to investigate the potential causal relationship between normal thyroid function and the intended outcomes.

### Outcomes (sarcopenia-related traits) datasets

We chose hand-grip strength and walking speed as measures of muscle function, and ALM as an indicator of muscle mass (3). Genetic instruments for hand-grip strength (n = 461,026), ALM (n = 450,243), and walking pace (n = 459,915) were obtained from the largest GWASs conducted by the UK Biobank with participants aged 48 to 73 years at the time of enrollment (21). The UK Biobank is an expansive biomedical database and a research resource. From 2006 to 2010, more than 500,000 participants aged between 40 to 69 years were recruited into the UK Biobank. Consenting participants completed a touchscreen-based questionnaire, face-to-face verbal interviews, physical measurements, provided information on their medical history and medication use, and biological samples were also collected. Follow-up for all participants involved linkage with hospital admissions data from England, Scotland, and Wales. Genetic association studies (GWAS) on sarcopenia-related variables were undertaken in European populations using data from population-based cohorts.

Hand-grip strength was quantified using a hydraulic hand dynamometer (Jamar J00105) designed to accommodate various hand sizes. ALM was evaluated by determining the total fat-free mass in the arms and legs via bioelectrical impedance analysis (BIA). The walking pace was classified according to self-reported responses, with “slow,” “steady/average,” and “brisk” being assigned numerical codes of 0, 1, and 2, respectively.

Data on the impact of different alleles, beta coefficients (β), standard errors (SE), and *p*-values for the variations associated with FT3, FT4, TT3, TT4, TSH, and FT3:FT4 ratio levels were gathered from each study for MR analyses.

### Statistical analyses

We employed the IVW method with multiplicative random effects as the primary approach for estimating the causal effect between exposure and outcome, which was considered to be the most reliable indicator in the absence of directional pleiotropy in the selected instrumental variables (IVs) (*p* for MR-Egger intercept > 0.05). The Cochran’s Q test was conducted to evaluate the presence of substantial heterogeneity among the selected independent variables. In the event that the heterogeneity is not statistically significant, it may be appropriate to utilize the fixed-effects model; however, if it is statistically significant, employing the IVW method with multiplicative random effects would be more suitable (22). In light of conducting multiple tests, we employed the Bonferroni method to adjust the level of significance by implementing a more stringent p-value threshold of 0.05/n (where n represents the number of independent hypotheses).

### Sensitivity analysis

To enhance the credibility of the MR causal effect estimation, we carried out multiple sensitivity analyses.

We initially employed the MR-Egger method to detect any potential pleiotropy of IVS, and the adjusted causal effect could be derived from the estimation for MR-Egger regression slope, as previously mentioned. The methodology utilized in this study exhibits low testing efficiency, thus we exclusively employed the MR-Egger method for the identification of pleiotropic effects, as opposed to evaluating causal effects (23).

We also utilized the weighted median method as an additional approach for sensitivity analysis to assess the reliability of the MR estimates, given its demonstrated advantages over the MR-Egger method. The weighted median method has been found to possess greater capacity in detecting causal effects and lower type I error, thus ensuring robustness in generating accurate estimates even when up to 50% of the SNPs are invalid IVs (24).

We employed MR- Pleiotropy RESidual Sum and Outlier (PRESSO) as a statistical method to identify and remove potential pleiotropic IVs. This approach assumes that at least 50% of the SNPs are valid, and can detect pleiotropy by examining outliers among the selected SNPs contributing to the MR estimate. Furthermore, MR-PRESSO is capable of adjusting estimates by eliminating outliers and evaluating the significant difference in causal effect estimation before and after outlier correction. In this study, adjusted estimates obtained from MR-PRESSO were considered the primary indicator of causal effect estimation in cases where horizontal pleiotropy was present (25).

We performed all statistical analyses using the Two-sample MR package within the R software environment.

## Results

### Influence of genetically predicted thyroid hormone on sarcopenia-related traits

The results did not suggest any potential causal relationship between sarcopenia-related traits and exposure to thyroid hormone as a contributing factor. We have identified 6, 65, 13,162, and 13 distinct genetic variants in linkage disequilibrium (LD)-independent (*r* ^2^ < 0.001 and kb = 10,000) that have reached the genome-wide significance threshold (*p* < 5×10^−8^) for FT3, FT4, TT3, TSH, and FT3:FT4 ratio levels respectively. The IVs were employed in the analysis of hand-grip strength, ALM, and walking pace. One set of IVs exhibited no significant heterogeneity (*p* > 0.05). The IVW fixed-effects model was utilized for those lacking heterogeneity, while the IVW random-effects model was applied to IVs demonstrating heterogeneity. We utilized a Bonferroni corrected significance threshold of 0.00333 (0.05/15) as previously mentioned. The IVW analysis revealed that there was no statistically significant causal relationship between genetically instrumented thyroid hormone and hand-grip strength, ALM, and walking pace, as the *p*-values did not reach the threshold for significance. The MR-Egger analysis did not reveal any evidence of directional pleiotropy for the selected IVs (**Table 1**). Consistent with the IVW results, both the Weighted median and MR-PRESSO estimates indicated the absence of a causal relationship between thyroid hormone and hand-grip strength (**Table 2**). Based on the aforementioned findings, there is no indication of a causal relationship between genetically predicted thyroid hormone levels and sarcopenia-related traits in the discovery cohort.

**Table 1:** Association of thyroid function with sarcopenia-related traits using MR-Egger and IVW analysis.

Exposures	Outcomes	No. of IVs	Heterogeneity test	MR Egger	IVW	
			Cochran’s Q (*p*)	Intercept (*p*)	Estimates (95% CI)	*p*
FT3	hand-grip strength	6	15.684 (0.003)	0.004 (0.569)	-0.057 (-0.098, -0.016)	0.007
FT3	Appendicular lean mass	6	57.97 (<0.001)	-0.007 (0.660)	0.034 (-0.062, 0.133)	0.505
FT3	walking pace	6	6.966 (0.138)	-0.004 (0.338)	-0.010 (-0.035, 0.016)	0.461
FT4	hand-grip strength	65	549.99 (<0.001)	-0.001 (0.315)	-0.010 (-0.037, 0.018)	0.494
FT4	Appendicular lean mass	65	1866.94 (<0.001)	0.001 (0.907)	-0.019 (-0.083, 0.045)	0.56
FT4	walking pace	65	393.964 (<0.001)	0.001 (0.991)	-0.007 (-0.026, 0.013)	0.512
TT3	hand-grip strength	13	44.080 (<0.001)	-0.002 (0.500)	0.488 (-0.036, 0.017)	0.488
TT3	Appendicular lean mass	13	74.680 (<0.001)	0.001 (0.861)	0.033 (-0.010, 0.076)	0.132
TT3	walking pace	13	27.091 (0.004)	-0.002 (0.244)	-0.004 (-0.022, 0.015)	0.692
TSH	hand-grip strength	162	599.42 (<0.001)	0.001 (0.479)	0.016 (0.004, 0.029)	0.008
TSH	Appendicular lean mass	162	1894.59 (<0.001)	0.001 (0.717)	0.024 (-0.004, 0.051)	0.09
TSH	walking pace	162	383.422 (<0.001)	-0.001 (0.635)	0.004 (-0.005, 0.012)	0.385
FT3/FT4 ratio	hand-grip strength	13	64.734 (<0.001)	-0.002 (0.403)	-0.114 (-0.268, 0.040)	0.147
FT3/FT4 ratio	Appendicular lean mass	13	496.463 (<0.001)	-0.006 (0.469)	0.133 (-0.408, 0.673)	0.631
FT3/FT4 ratio	walking pace	13	56.06 (<0.001)	0.002 (0.727)	-0.013 (-0.131, 0.107)	0.837

Values with p < 0.00333 denote statistical significance. MR, Mendelian randomization; IVW, inverse variance weighted; IVs, instrumental variables; CI, confidence interval.

**Table 2:** Association of thyroid function with sarcopenia-related traits using weighted median and MRPRESSO analysis.

Exposures	Outcomes	Weighted median			MR-PRESSO			
		No. of IVs	Estimates (95% CI)	*p*	No. of IVs	Estimates (95% CI)	*p*	IVs outlier
FT3	hand-grip strength	6	-0.048 (-0.084, -0.012)	0.009	6	-0.062 (-0.133, 0.009)	0.087	NA
FT3	Appendicular lean mass	6	0.075 (0.033, 0.118)	0.001*	6	0.051 (-0.049, 0.151)	0.318	NA
FT3	walking pace	6	-0.020 (-0.047, 0.007)	0.142	6	-0.010 (-0.039, 0.019)	0.494	NA
FT4	hand-grip strength	65	0.003 (-0.017, 0.022)	0.787	53	-0.006 (-0.022, 0.010)	0.452	rs1243192, rs12878623, rs13107325, rs225014, rs4842131, rs6430552, rs76895963, rs775738, rs7858917, rs9356988, rs951366, rs965513
FT4	Appendicular lean mass	65	-0.032 (-0.056, -0.009)	0.006	44	-0.022 (-0.086, 0.042)	0.5	rs10822059, rs10838738, rs11626434, rs178659, rs225014, rs35311109, rs3821083, rs4842131, rs56069042, rs6510177, rs6910649, rs7096254, rs75705948, rs76895963, rs785490, rs7858917, rs8002440, rs8038670, rs881858, rs9496614, rs951366
FT4	walking pace	65	-0.007 (-0.020,0.007)	0.342	58	-0.005 (-0.017, 0.007)	0.413	rs11626434, rs1243192, rs13107325, rs6430552, rs6839, rs7146111, rs794375
TT3	hand-grip strength	13	0.003 (-0.019, 0.025)	0.8	11	-0.004 (-0.025, 0.017)	0.709	rs28929474, rs925488
TT3	Appendicular lean mass	13	0.013 (-0.013, 0.039)	0.335	12	0.017 (-0.011, 0.045)	0.234	rs61987066
TT3	walking pace	13	-0.011 (-0.029, 0.990)	0.251	12	-0.009 (-0.024, 0.006)	0.243	rs112301964
TSH	hand-grip strength	162	0.013 (0.002, 0.024)	0.02	147	0.015 (0.003, 0.027)	0.015	rs1129735, rs11641216, rs1203921, rs1690789, rs1755770, rs199528, rs30232, rs568995, rs6414089, rs677355, rs72682416, rs7310615, rs7589228, rs9264916, rs9889941
TSH	Appendicular lean mass	162	0.013 (-0.004, 0.030)	0.13	126	0.021 (-0.006, 0.048)	0.129	rs10504444, rs17197194, rs17729624, rs179785, rs30232, rs35717482, rs4273585, rs440225, rs4933466, rs568995, rs6140066, rs62621812, rs6414089, rs6567094, rs677355, rs7529705, rs7589228, rs7955258, rs11602347, rs7310615, rs10739496, rs10878984, rs1114707, rs1129735, rs13197926, rs1385737, rs1690789, rs2031365, rs55717031, rs58722186, rs5997969, rs6724363, rs751171, rs7675216, rs78738909, rs879736
TSH	walking pace	162	0.005 (-0.004, 0.015)	0.283	157	0.005 (-0.002, 0.012)	0.172	rs1129735, rs11641216, rs11670562, rs1875057, rs6494466
FT3/FT4 ratio	hand-grip strength	13	-0.035 (-0.135, 0.065)	0.494	9	-0.177(-0.376, 0.022)	0.082	rs11675342, rs4115668, rs11626434, rs6553487
FT3/FT4 ratio	Appendicular lean mass	13	0.195 (0.080, 0.310)	0.001*	8	0.196 (0.028, 0.364)	0.022	rs2235544, rs11626434, rs3821083, rs7077440, rs925489
FT3/FT4 ratio	walking pace	13	0.016 (-0.096, 0.064)	0.699	11	0.039 (-0.027, 0.105)	0.249	rs11626434, rs11675342

*p < 0.00333. MR, Mendelian randomization; PRESSO, Pleiotropy RESidual Sum and Outlier; IVs, instrumental variables; CI, confidence interval; NA, not applicable.

### Influence of genetically predicted sarcopenia-related traits on thyroid hormone

The results did not demonstrate a direct association between sarcopenia-related traits and thyroid hormone. We have identified 79, 555, and 56 LD-independent (*r* ^2^ < 0.001 and kb = 10,000) SNPs that have reached the genome-wide significance level (*p* < 5×10^−8^) as potential instrumental variables for hand-grip strength, ALM, and walking pace. The heterogeneity test revealed a significant diversity among the selected instrumental variables (IVs) (*p* < 0.05). Consequently, the MR analyses employed the IVW method with multiplicative random effects. However, the IVW analysis did not identify any significant causal relationship between genetically instrumented sarcopenia-related traits and thyroid hormone levels. The MR-Egger analysis revealed the presence of directional pleiotropy in instrumental variables for ALM and FT3/FT4 ratio (**Table 3**). However, upon adjustment for directional pleiotropy using MR-PRESSO, no significant causal relationship between genetically instrumented ALM and FT3/FT4 ratio was observed. The findings remained consistent when employing the Weighted median method (**Table 4**). Based on the aforementioned results, there is no indication of a causal association between sarcopenia-related traits and thyroid hormone levels.

**Table 3:** Association of sarcopenia-related traits with thyroid function using MR-Egger and IVW analysis.

Exposures	Outcomes	No. of IVs	Heterogeneity test	MR Egger	IVW	
			Cochran’s Q (*p*)	Intercept (*p*)	Estimates (95% CI)	*p*
hand-grip strength	FT3	79	147.060 (<0.001)	0.001 (0.643)	-0.018 (-0.091, 0.053)	0.608
hand-grip strength	FT4	79	336.542 (<0.001)	0.001 (0.756)	-0.091 (-0.171, -0.011)	0.026
hand-grip strength	TT3	79	103.166 (0.025)	0.003 (0.473)	-0.052 (-0.171, 0.067)	0.39
hand-grip strength	TSH	79	287.088 (<0.001)	0.002 (0.367)	0.047 (-0.004, 0.097)	0.069
hand-grip strength	FT3/FT4 ratio	79	105.418 (0.017)	0.001 (0.882)	0.001 (-0.011, 0.012)	0.089
Appendicular lean mass	FT3	555	668.587 (<0.001)	0.001 (0.106)	-0.040 (-0.069, -0.012)	0.005
Appendicular lean mass	FT4	555	1340.846 (<0.001)	-0.001 (0.062)	0.008 (-0.022, 0.037)	0.606
Appendicular lean mass	TT3	555	551.729 (0.507)	0.001 (0.780)	-0.062 (-0.113, -0.011)	0.017
Appendicular lean mass	TSH	555	2871.931(<0.001)	-0.001 (0.066)	0.003 (-0.026, 0.033)	0.822
Appendicular lean mass	FT3/FT4 ratio	555	821.972 (<0.001)	0.001 (0.008)	-0.006 (-0.012, -0.001)	0.046
walking pace	FT3	56	102.986 (<0.001)	0.001 (0.786)	-0.082 (-0.342, 0.178)	0.537
walking pace	FT4	56	298.892 (<0.001)	0.009 (0.154)	-0.036 (-0.365, 0.293)	0.83
walking pace	TT3	56	83.105 (0.007)	-0.005 (0.614)	-0.228 (-0.690, 0.235)	0.335
walking pace	TSH	56	158.174 (<0.001)	-0.001 (0.918)	0.065 (0.095, 0.226)	0.426
walking pace	FT3/FT4 ratio	56	102.117 (<0.001)	0.001 (0.979)	-0.026 (-0.075, 0.024)	0.309

Values with p < 0.00333 denote statistical significance. MR, Mendelian randomization; IVW, inverse variance weighted; IVs, instrumental variables; CI, confidence interval.

**Table 4:** Association of sarcopenia-related traits with thyroid function using weighted median and MRPRESSO analysis.

Exposures	Outcomes	Weighted median			MR-PRESSO			
		No. of IVs	Estimates (95% CI)	*p*	No. of IVs	Estimates (95% CI)	*p*	IVs outlier
hand-grip strength	FT3	79	-0.025 (-0.115, 0.065)	0.588	76	-0.021 (-0.086, 0.045)	0.535	rs1167827, rs1516725, rs6567160
hand-grip strength	FT4	79	-0.041 (-0.116, 0.034)	0.288	71	-0.055 (-0.110, 0.001)	0.054	rs11030104, rs1167827, rs13107325, rs3817334, rs3888190, rs543874, rs6567160, rs9304665
hand-grip strength	TT3	79	-0.100 (-0.272, 0.072)	0.253	79	-0.052 (-0.172, 0.067)	0.392	NA
hand-grip strength	TSH	79	0.01 (-0.033, 0.053)	0.64	73	0.045 (0.011, 0.077)	0.009	rs1167827, rs12286929, rs1460676, rs3888190, rs7550711, rs879620
hand-grip strength	FT3/FT4 ratio	79	0.004 (-0.014, 0.021)	0.674	77	0.001 (-0.010, 0.010)	0.997	rs13107325, rs3888190
Appendicular lean mass	FT3	555	-0.032 (-0.075, 0.010)	0.136	550	-0.040 (-0.067, -0.013)	0.004	rs1260326, rs2142331, rs2812208, rs3792819, rs59950280
Appendicular lean mass	FT4	550	0.017 (-0.017, 0.052)	0.328	530	0.005 (-0.022, 0.032)	0.718	rs10858246, rs10948, rs11233117, rs12074850, rs17773965, rs244711, rs4655345, rs4752829, rs55852614, rs6054390, rs7107356, rs7228151, rs7328187, rs73384223, rs76895963, rs78378222, rs78766798, rs9391254, rs951366, rs9894577
Appendicular lean mass	TT3	555	-0.054 (-0.141, 0.033)	0.227	555	-0.065 (-0.113, -0.011)	0.011	NA
Appendicular lean mass	TSH	555	0.001 (-0.023, 0.025)	0.933	517	0.009 (-0.009, 0.027)	0.335	rs10824307, rs10858246, rs11121615, rs12074850, rs12512942, rs12533452, rs12563442, rs13391980, rs1478575, rs17773965, rs1797070, rs2005172, rs2019203, rs2240735, rs246177, rs2754255, rs2763263, rs2871865, rs3184504, rs3205136, rs3768495, rs40270, rs4244809, rs4287835, rs4752689, rs55852614, rs62621812, rs7129320, rs7137546, rs7228151, rs73384223, rs7633464, rs76895963, rs772222, rs7768382, rs8042578, rs900399, rs905938
Appendicular lean mass	FT3/FT4 ratio	550	-0.005 (-0.014, 0.001)	0.233	543	-0.005 (-0.011, 0.001)	0.091	rs147233090, rs4655345, rs55852614, rs655113, rs7107356, rs713467, rs78000963
walking pace	FT3	56	-0.001 (-0.301, 0.299)	0.997	55	-0.112 (-0.358, 0.134)	0.372	rs6763292
walking pace	FT4	56	0.266 (0.024, 0.508)	0.031	50	0.113 (-0.027, 0.253)	0.113	rs113825410, rs12747822, rs205262, rs4839898, rs10750025, rs11077815
walking pace	TT3	56	-0.094 (-0.690, 0.503)	0.758	56	-0.231 (-0.688, 0.266)	0.322	NA
walking pace	TSH	56	0.105 (-0.062, 0.271)	0.218	54	0.103 (-0.047, 0.253)	0.177	rs11548200, rs11150623,
walking pace	FT3/FT4 ratio	56	-0.061 (-0.117, -0.005)	0.032	555	-0.040(-0.087, 0.007)	0.092	rs205262

MR, Mendelian randomization; PRESSO, Pleiotropy RESidual Sum and Outlier; IVs, instrumental variables; CI, confidence interval; NA, not applicable.

## Discussion

In this study, we performed a bi-directional two-sample MR analysis to investigate the causal relationships between susceptibility to various thyroid hormone levels and sarcopenia-related traits. Using this approach, we did not observe any significant correlation between susceptibility to thyroid hormone and hand-grip strength, ALM, and walking pace. This suggests that the reported associations in epidemiological studies may be confounded by unmeasured variables or common genetic factors. We performed multiple sensitivity analyses to differentiate between a true negative result and potential lack of validity in the MR studies, ensuring that the MR assumptions were satisfied. Our consistent findings across various methods provide us with confidence in the robustness of our MR analyses, indicating no substantial causal effects of the exposures on the outcomes.

Understanding the correlation between thyroid hormone and sarcopenia is essential for the management and prevention of these conditions, given their substantial implications for human health (26). A study conducted by Sheng et al. revealed evidence supporting a correlation between lower FT3 levels within the normal range and an elevated risk of sarcopenia in elderly individuals (27). In addition, Li et al. investigated TT3, FT3, TT4, FT4, TSH, rT3, TBG levels and their association with hand-grip strength and gait speed. The findings indicated a positive relationship between FT3, TT3 and hand-grip strength as well as gait speed. However, after adjusting for various factors including age, gender, BMI, physical activity level, FRIAL scores, ALT and sCr levels; it was observed that TT3 rather than FT3 exhibited a significant association with sarcopenia and hand-grip strength (28). The results do not align with the findings of Sheng et al’s study. One potential explanation for these inconsistencies is that the participants in each study were at different life stages. Age can exert a significant influence on thyroid hormone levels, particularly FT3, as well as on the levels of deiodinase and TBG, which directly impact FT3 levels (6, 29). The FT3:FT4 ratio serves as an indicator of the conversion rate from FT4 to FT3. As suggested by Hata S et al., assessments of the FT3/FT4 ratios could potentially serve as significant indicators for muscle mass and strength in male individuals, thus highlighting its potential relevance in clinical research (30). The study presents findings that are inconsistent with the results of our research. Despite the abundance of epidemiological studies, including those mentioned previously, examining the association between thyroid function and sarcopenia, there remains ongoing debate regarding this relationship primarily due to potential selection bias, residual confounding, and reverse causality in observational studies, which complicates the ability to establish causal conclusions. Our analysis, utilizing two samples, yielded a more robust and potentially broadly applicable assessment of the causal relationship between thyroid hormone and sarcopenia.

The regulation of skeletal muscle function is governed by a complex system involving signaling from the neuroendocrine system (31). Research has indicated a correlation between thyroid hormone and the initiation of sarcopenia. These findings suggest that the development of sarcopenia may be impacted by dysfunction in the thyroid hormone system (26, 27, 32). The changes observed in skeletal muscle during the aging process bear resemblance to the modifications associated with a decrease in thyroid hormone signaling (33, 34). It remains unclear whether variations in thyroid hormone levels are a consequence of sarcopenia or if they play a role in its progression. While no direct genetic link between thyroid hormone and sarcopenia-related traits was identified in this study, further research is needed to fully understand the association between thyroid hormone and sarcopenia.

Our study has several limitations. Since it was conducted in Europe, the generalizability of our findings to other populations may be limited. Additionally, old age and an individual’s specific health condition are profoundly intertwined with thyroid hormone and development of sarcopenia. Regrettably, we were precluded from investigating these variables in greater depth owing to the unavailability of individual-level data. Finally, although evidence from our study did not support a causal association thyroid hormone and sarcopenia-related traits, we could not completely rule out the possibility that the effect size might be relatively small, although the sample size of our study was large enough to detect this difference, however we did not perform subgroup analysis according to the age and sex in the present research.

## Conclusions

Our research results showed that there were no causal relationships between thyroid hormone and sarcopenia-related traits at the genetic level. However, that does not rule out the possibility that they are related on a level other than genetic. Deeper and more extensive research is needed to investigate these possible links.

## Data Availability

The original contributions presented in the study are included in the article/supplementary material. Further inquiries can be directed to the corresponding author.
